# Prevalence of *Acinetobacter baumannii* in Saudi Arabia: risk factors, antimicrobial resistance patterns and mechanisms of carbapenem resistance

**DOI:** 10.1186/s12941-018-0301-x

**Published:** 2019-01-03

**Authors:** Mutasim E. Ibrahim

**Affiliations:** 1 Department of Basic Medical Science, College of Medicine, University of Bisha, Bisha, Saudi Arabia; 2 Unit of Medical Microbiology, College of Medicine, University of Bisha, P. O. Box 731, Bisha, 61922 Saudi Arabia

**Keywords:** *A. baumannii*, Risk factors, Antimicrobial susceptibility, Carbapenem resistance mechanisms, Saudi Arabia

## Abstract

*Acinetobacter baumannii* is an important opportunistic pathogen due to its capabilities for developing mechanisms of resistance to a wide range of antimicrobial agents including carbapenems. This review described the risk factors, antimicrobial susceptibility and mechanisms of carbapenem resistance of *A. baumannii* from different geographical regions of Saudi Arabia. Several factors including complexity of intensive care unit (ICU) environments, increased numbers of patients with serious diseases, wide spread gastrointestinal colonization and extensive use of antimicrobial drugs led to a wide prevalence of *A. baumannii* infections in hospitals in Saudi Arabia. *A. baumannii* has been noted to be less susceptible to antimicrobials agents, including carbapenems, over time, resulting in the evolution of multidrug-resistant (MDR) strains. Dissemination of MDR *A. baumannii* is attributed to the extreme use of wide-spectrum antimicrobial drugs in hospitals, cross infection between inpatients, invasive ICU procedures, and hospitalized patients with diabetic and cancer those are under frequent invasive diagnostic and therapeutic interventions. Although an increasing prevalence of colistin and tigecycline resistance has been reported in many hospitals, combinations of these agents with carbapenems or other antibiotics remain the best therapeutic choice and reasonably safe to treat patients with MDR *A. baumannii* infections. The wide distribution of carbapenem resistant *A. baumannii* (CRAB) due to several mechanisms with diverse genetic determinants has been documented. Although OXA-23 β-lactamase and OXA-51 β-lactamase are the most common genes responsible for CRAB, other novel genes such as blaVIM, PER-1-like and GES-5 have been discovered in carbapenem resistant strains. The high rates of MDR *A. baumannii* in Saudi hospitals indicate that extensive investigation into the molecular basis of MDR and developing new therapies of CRAB is needed. Moreover, the development of a local antibiogram database coupled with a nationwide antimicrobial stewardship and infection prevention program might help to improve our knowledge of the resistance patterns of *A. baumannii,* and in developing a treatment protocol for decreasing the infection burden in Saudi Arabia.

## Background

Over the last few years, *Acinetobacter baumannii* (*A. baumannii*) has become an important opportunistic pathogen that is widespread in hospitals and other healthcare settings [1–3]. It has the ability to colonize a large variety of surfaces and can survive for prolonged periods under a wide range of environmental conditions [4]. The emergence of *A. baumannii* strains resistant to broad-spectrum antimicrobial agents in hospital settings has become a major health burden, due to the limited treatment options for infections caused by this pathogen [2, 4, 5]. *A. baumannii* has to capability to develops resistance mechanisms to a wide range of antimicrobial agents, including carbapenems [2, 6, 7]. The increasing rates of resistance to carbapenems and other therapeutic options such as colistin and polymyxins is alarming, and have become a health issue of global concern [8, 9]. The patterns of the spread of resistance is facilitated by complex factors, including the presence of mobile genetic elements, the misuse of antimicrobial drugs, poor infection control practices, and increased international travel [10, 11]. Recognizing such factors might contribute to the identification of innovative therapeutic targets and lead to improvements in the outcomes of infections caused by this bacterium [12–14]. Several studies from the Gulf Cooperation Council, including in Saudi Arabia, have reported increasing carbapenem resistance among *A. baumannii* isolates [6, 15]. Following the detection of the first OXA-type carbapenemases in 1985 in a *A. baumannii* isolate, many new types have been discovered and the number of OXA-type b-lactamases is increasing exponentially [16, 17]. This article reviewed the epidemiology, risk factors and patterns of antimicrobial resistance in *A. baumannii* among different regions of Saudi Arabia and focused on the mechanisms of carbapenem resistance.

## Epidemiology and risk factors

*Acinetobacter baumannii* is able to cause a spectrum of infections including, lower respiratory tract, blood, urinary tract and wound infections [1, 12, 18]. Numerous studies in Saudi Arabia have assessed risk factors for infections caused by *A. baumannii* [9, 18–22]. The presence of *A. baumannii* infection or colonization has been investigated among inpatients, healthcare workers and environmental sites in intensive care units (ICU) [22]. A study in 2001 reported the dissemination of endemic *A. baumannii* strains among ICU patients receiving mechanical ventilation, and tracheal secretions have been found to be a site of colonization among these patients [21]. In February 2013, *A. baumannii* was also identified during an outbreak in the adult ICU of a tertiary hospital in Riyadh. Senok et al. [14] found that *A. baumannii* was widely disseminated in the ICU environment, including on a computer mouse and a stethoscope which suggests that healthcare workers may be involved in the transmission of these clones within the ICU. In order to avoid such outbreaks, regimens of enhanced hand washing, staff education and environmental decontamination of critical care areas in hospitals need to be established [14, 21]. El-Ageery et al. [22] reported that the risk factors for the infection were mechanical ventilation, surgical interventions, and the presence of other co-morbid conditions. The study revealed a causal link between a contaminated ventilator and subsequent multidrug-resistant (MDR) *A. baumannii*. Studies have found that ICU admission is an important risk factor for ventilator-associated pneumonia caused by *A. baumannii*, and the risk of infections increased among patients over 60 years and those who spent a prolonged time on artificial ventilation [19, 23]. Indeed older patients are more susceptible to infections due to many factors including, impairment of structure and function of certain body systems and decreasing of immune response that induce other conditions like, catheterization, malnutrition, prolonged hospital stay [19, 24]. In addition, another study found that the risk of infection due non-fermenting gram-negative nosocomial pathogens, including *A. baumannii,* increased with ICU admission, increase patient’s age, the use of catheters or intubation, prior surgery and prolonged hospital admission [25]. Ballouz et al. [26] indicated that a prolonged hospital admission is frequently associated with reduction of functionality, and increased exposure to steroids and antibiotics; and these factors seem to impact the mortality of *A. baumannii* bacteremia. A hospital-based, matched case–control study found that the risk factors associated with carbapenem resistant *A. baumannii* (CRAB) in nosocomial infections in ICU include immunosuppression, invasive procedures, central venous catheters, and an endotracheal intubation [7]. Gastrointestinal colonization by *A. baumannii* in ICU patients is a significant risk factor for spreading antimicrobial resistance, increases disease severity and provides an important reservoir for clinical infections and hospital outbreaks [27]. Reducing the risk factors for *A. baumannii* infection necessitates essential monitoring steps such as screening high risk patients for colonization, application of ventilator care bundles, shortened duration of ventilation and encouraging oral-gastric rather than nasal-gastric intubation [23, 27, 28]. One study indicates that mass gathering during pilgrimage (Hajj) season in Holy Makkah is a potential risk for acquiring and transmitting MDR *A. baumannii.* In 2014, pharyngeal and rectal swab samples were collected from 98 pilgrims before and after they traveled to the Hajj to investigate the acquisition of MDR bacteria. *A. baumannii* strains were isolated from 16 pharyngeal swab samples and 26 post-Hajj rectal swab samples; therefore, personal hygiene should be encouraged and monitored to avoid MDR bacterial transmission while performing such rituals in Makkah [29].

The patterns of resistance and severity of *A. baumannii* infection depend upon the patient’s susceptibility to infection as a result of their underlying diseases [30, 31]. Patients with underlying illness such as DM, cancer, renal impairment and chronic pulmonary disorders have immune deficits that lead to increased susceptibility to infection caused by opportunistic pathogens [7, 9, 32]. However, individuals with such conditions tend to become at long-term hospitalization and under frequent invasive diagnostic and therapeutic interventions [33]. Numerous studies indicated that the severity of underlying disease, broad-spectrum antimicrobial use, prolonged hospitalization, ICU stays, the presence of invasive devices, and prolonged mechanical ventilation were risk factors for acquisition of multi-drug resistant *A*. *baumannii* strains [7, 25, 34]. According to Al-anazi and Al-Jasser, [9] patients with hematological malignancies and recipients of various types of hematopoietic stem cell transplants are at high risk of developing infectious complications related to *A. baumannii*. In a retrospective study of 61 episodes of gram-negative bacteria occurring in 56 patients with malignancies at King Khalid University Hospital, Riyadh, *A. baumannii* accounted for 18% of infections. The elevated resistance rate among the pathogens isolated from patients with malignancies recommends testing for antimicrobial sensitivity prior to starting antimicrobial treatment [35]. Clinical data from Saudi Arabia and throughout the world indicated that individuals with diabetes are at greater risk of acquiring a serious *A. baumannii* infection than the rest of the population [6, 36]. In a descriptive study of 72 patients from hospitals in Makkah and Jeddah Cities infected with *A. baumannii*, 36% had underlying diseases with diabetes mellitus (11%) being the most common [31]. Between 2006 and 2007, clinical samples were collected from different sites of infection of hospitalized and community patients at major hospitals and medical center across Saudi Arabia, and the *A. baumannii* isolates were all highly resistant to the most common antimicrobial agents [37]. However, all the isolates were susceptible to imipenem, colistin, but revealed low resistance rates to meropenem (20%). This study suggested that carbapenem resistance in *A. baumannii* might be increased in certain body areas in diabetic patients [37], and another study indicated that diabetic patients were significantly more likely to carry CRAB isolates in general [6]. Clonal spread of CRAB among diabetic patients with pneumonia and wound infections has been widely reported in different hospitals and medical centers in Saudi Arabia [22, 31, 38]. Moreover, previous studies in the country demonstrated that diabetics were infected with specific clones of *A. baumannii* that differ from those found in non-diabetic patients. The high levels of multi-resistance in carbapenems in *A. baumannii* responsible for deep-sited infections of diabetic patients were due to the co-existence and acquisition of diverse carbapenem-resistance genes [6, 16, 37]. Saudi Arabia is one of the highest prevalence of diabetes among the adult population in the world and 19% of them developed foot ulcers [39]. It is widely accepted that individual develops foot ulcer infections due to impaired glycemic control and low immune status and most likely being hospitalized [40, 41]. The emergence of CRAB among patients with diabetic foot ulcers is facilitated by a number of factors including inappropriate antibiotic treatment, the chronic nature of the wound, the frequency of hospital admission and the presence of mobile genetic elements [42]. Furthermore, peripheral artery diseases associated with diabetic foot ulcers results in poor penetration of antibiotics into the lower limb tissues, thereby promoting selection of resistant bacterial strains [43].

## Emergence of antimicrobial resistance

Resistance in *A. baumannii* to broad-spectrum antimicrobial agents in hospital settings is now an emerging issue worldwide [44]. Surveillance studies indicate that the rates of resistance to antimicrobial agents among *A. baumannii* are increasing in South east Asia [45], the Arabian Peninsula [15] and in many parts of the globe [44]. The presence of nosocomial MDR *A. baumannii* in several Saudi Arabia hospitals have become a significant healthcare and economic issue [18, 46, 47]. Saudi Arabia is divided into 13 administrative regions, and geographically these regions are distributed in five major areas of the country (central, eastern, northern, southern and western areas) [48]. Most of the studies about *A. baumannii* antimicrobial resistance were conducted in the central, western and eastern areas and small data came from the southern and northern areas (Table 1). Most of these findings confirmed that *A. baumannii* was becoming increasingly resistant to different types of antimicrobial agents.

**Table 1:** Resistance rates of *A. baumannii* to different antimicrobial classes collected from hospitals of different geographical areas in Saudi Arabia

Area	Region	Refs.	Year of survey	Penicillins			Cephalosporin’s				Carbapenems		
				AMC	PIP	PIP-TAZ	CFN	CFM	CAZ	CTX	AZO	IMP	MER
Central	Qassim	[47]	2011	–	–	42	–	67	89	–	–	9	–
	Qassim	[49]	2014–2015	100	100	–	–	–	100	–	–	–	–
	Riyadh	[50]	2006–2008	–	–	–	–	98.8	–	99.2	–	79.1	92.1
	Riyadh	[2]	2006–2014	–	–	–	–	96.9	96.2	95.4	–	76.9	88.5
	Riyadh	[51]	2009	96	–	93	–	–	88	–	83	89	91
	Riyadh	[52]	2010	–	–	–	–	78	78	–	78	89	89
	Riyadh	[53]	2011	–	–	–	–	100	100	–	100	63.6	
	Riyadh	[18]	2006	–	–	–	–	–	75	–	–	36	19
			2012	–	–	–	–	–	83	–	–	91.7	89
	Riyadh	[35]	2013–2015	–	–	–	–	67.7	91.9	–	–	72.7	81.8
West	Jeddah	[54]	2012	–	–	–	–	27	25	–	–	66	–
	Jeddah	[1]	1999–2000	71	60	–	–	–	59	66	81	15	–
	Makkah and Jeddah	[31]	2011	–	93.1	–	–	72.2	69.4	75	80.5	62.5	–
	Madinah	[55]	2014	93.7	70.8	–	100	–	100	–	95.8	–	–
	Madinah	[22]	6 month	100	–	100	100	100	100	100	100	100	100
	Makkah	[56]	2004–2005	87	73	8	92	45	79	–	90	14	–
	Makkah	[57]	2005–2006	92.9	–	–	97.9	–	86.9	83.3	95.3	45.9	28
	Makkah	[58]	2015	–	–	–	–	77	77	100	–	90	64
East	Dammam	[12]	2010–2012	–	–	33.3	–	73.8	85.1	–	–	32.6	33.3
	Dammam	[27]	2014	–		–	–	100	–	–	–	–	–
	Dammam and Dhahran	[59]	1998–2004	–	–	–	89	–	38	–	–	3	–
	Dammam and Khobar	[60]	2015	–	–	–	–	67.7	71	87	–	–	–
	Dammam	[61]	2014	–	–	–	–	100	100	100	–	100	100
South	Aseer	[62]	2011–2012	–	–	–	–	–	39	–	–	3	–
	Aseer	[63]	2013–2014	–	81.5	–	–	89.8	–	–	–	51.9	50
	Aseer	[19]	2014–2015	–	97.1	–	–	96.6	92.7	97.8	92	95.5	100
	Najran	[25]	2012–2013	–	–	32.4	–	45.6	91.2	–	–	7.4	1.5

Area	Region	Refs.	Year of survey	Aminoglycosides			CIP	SXT	CL	TIG
				AK	GN	TOB				
Central	Qassim	[47]	2011	63	51	–	90	66	–	–
	Qassim	[49]	2014–2015	80	90.9	–	100	100	–	–
	Riyadh	[50]	2006–2008	58.1	96.4	41.5	99.6	84.4	3.0	–
	Riyadh	[2]	2006–2014	56.9	93.8	40	96.2	99.2	1.5	–
	Riyadh	[51]	2009	86	80	–	92	56	0.0	43
	Riyadh	[52]	2010	37	52	–	93	–	30	56
	Riyadh	[53]	2011	40	–	–	94.5	–		45.5
	Riyadh	[18]	2006	71	–	–	54	–	0.0	–
			2012	83	–	–	89.2	–	0.0	–
	Riyadh	[35]	2013–2015	72.7	54.5	–	91.9	–	–	–
West	Jeddah	[54]	2012	34	57	–	27	–	8	–
	Jeddah	[1]	1999–2000	74	56	–	38	54	–	–
	Makkah and Jeddah	[31]	2011	40.3	51.4	–	69.4	72.2	–	–
	Madinah	[55]	2014	89.6	81.2	–	100	–	–	–
	Madinah	[22]	6 month	89	94.5	–	94.5	93.3	–	0.0
	Makkah	[56]	2004–2005	75	72	–	77	76	–	–
	Makkah	[57]	2005–2006	84.2	76.2	56.5	–	75.4	–	–
	Makkah	[58]	2015	67	46	50	83	–	–	–
East	Dammam	[12]	2010–2012	27	54.6	–	69.5	63.1	0.0	30
	Dammam	[27]	2014	40	62.9	–	100	–	0.0	51.4
	Dammam and Dhahran	[59]	1998–2004	34	26	25	30	23	–	–
	Dammam and Khobar	[60]	2015	25.8	29	–	74.2	–	0.0	–
	Dammam	[61]	2014	90	80	50	100	–	0.0	80
South	Aseer	[62]	2011–2012	33	25	24	30	–	0.0	–
	Aseer	[63]	2013–2014	75	81.5	79.6	82.9	65.7	4.6	–
	Aseer	[19]	2014–2015	83.7	94.5	93.2	93.9	25.5	0.0	–
	Najran	[25]	2012–2013	41.2	60.3	–	45.6	77.9	0.0	–

*AK* amikacin, *AMC* amoxicillin–clavulanic acid, *AZO* aztreonam, *CAZ* ceftazidime, *CFM* cefepime, *CFN* cefoxitin, *CIP* ciprofloxacin, *CL* colistin, *CTX* cefotaxime, *CXM* cefuroxime, *GN* gentamicin, *IMI* imipenem, *MERO* meropenem, *PIP-TAZO* piperacillin–tazobactam, *SXT* trimethoprim sulfamethoxazole, *TIG* tigecycline, *TOB* tobramicin

## Central area

Several studies have tested the sensitivity of *A. baumannii* from Riyadh to a wide range of antimicrobial classes. Among 253 *A. baumannii* isolates collected between 2006 and 2008 from patients at King Abdulaziz Medical City hospital in Riyadh, 92.1% were resistant to meropenem and 79.1% were resistant to imipenem [50]. Another study found that 96% of *A. baumannii* were resistant amoxicillin/clavulanic acid, 93% were resistant to piperacillin/tazobactam and 92% were resistant to ciprofloxacin [51]. During 2010, twenty-seven isolates of *A. baumannii* collected from different patients in a tertiary hospital were found to be highly resistant (78%) to ceftazidime, cefepime, and aztreonam. Of these isolates, 24 (89%) were resistant to one or more carbapenem (imipenem and meropenem), 15 (56%) were resistant to tigecycline and eight (30%) were resistant to colistin [52]. However, colistin and tigecycline combinations with carbapenems and other antibiotics are widely used as the best therapeutic options with high safety profile to treat MDR *A. baumannii* infections [64]. Such elevated resistance rate against tigecycline and colistin is of great concern since they are widely used for as a limited treatment option for MDR *A. baumannii* infections in Saudi Arabia [53, 65].

Several studies from the central region of Saudi Arabia have reported that the resistance rates of *A. baumannii* to different antimicrobial classes are increasing over time. A retrospective study conducted at Riyadh Military Hospital during a 6-year period from 2005 to 2010 suggests an increase in multiple resistant patterns among *A. baumannii* isolates from blood culture isolates [66]. Consistently, another study during a period from 2004 to 2009 at the adult ICU of King Fahad National Guard Hospital have been reported significant decreasing of susceptibility of *A. baumannii* to divers antimicrobial classes, including imipenem, meropenem, ciprofloxacin, and amikacin [67]. Likewise, Al-Obeid et al. [18] examined antimicrobial susceptibility of 506, 510 and 936 duplicate isolates of *A. baumannii* collected from a security forces hospital during 2006, 2009 and 2012, respectively. The resistance rates of *A. baumannii* to a variety of antimicrobial agents had changed by the following amounts: 19% (2006) to 89% (2012) for meropenem, 36% (2006) to 91.7% (2012) for imipenem, 54% (2006) to 89.2% (2012) for ciprofloxacin, 71% (2006) to 83% (2012) for amikacin, and 75% (2006) to 83% (2012) for ceftazidime. These high levels of resistance rates attributed to the excessive misuse of meropenem and imipenem and the reduction in the use of amikacin and gentamicin for the treatment of patients with *A. baumannii* infection. Therefore, guidelines for the management of the infection control practices to reduce spreading of MDR *A. baumannii* in hospital settings are needed [18].

Qassim is the second most populous region, located about 300 km north of Riyadh. A survey of the antimicrobial resistance patterns of clinical isolates was conducted over 1 year (2011) in a major hospital in Buraidah, a city in the Qassim region. *A. baumannii* had high rates of resistance to all of the antimicrobials tested, such as 90% to ciprofloxacin, 89% to ceftazidime, and 66% to trimethoprim/sulfamethoxazole [47]. In December 2014 to March 2015, a study was conducted in Al-Rass General Hospital in Al-Rass city to determine antimicrobial susceptibility of *A. baumannii* clinical isolates. Interestingly, *A. baumannii* isolates were highly resistant to all tested antimicrobial agents, such as amoxicillin/clavulanic acid (100%), ciprofloxacin (100%), piperacillin (100%), ceftazidime (100%), trimethoprim/sulfamethoxazole (100%), gentamicin (90.9%) and amikacin (80%) [49]. These figures were escalating that necessitate contentious monitoring of antimicrobial susceptibility along with appropriate infection control measure [47].

## Western area

In the western area, the Makkah region includes the two largest cities (Makkah and Jeddah) with the highest population outside the central region. Millions of Muslims from across the globe arrive annually in Makkah to perform pilgrimage and Umrah. These mass gatherings could be a good environment for spreading multi-drug resistant organisms around the world [29, 58]. In 2015, a study examined antimicrobial susceptibility patterns of bacterial pathogens recovered from pilgrims visiting emergency care departments in Makkah. *A. baumannii* was found to be highly resistant to cefotaxime (100%), cefepime and ceftazidime (77% each), ciprofloxacin (83%), imipenem (90%) and amikacin (67%) [58]. These high rates indicate a need to optimize and inspect the use of antibiotics when managing community-acquired infections among pilgrims from different parts of the world [58]. During a period from 2004 to 2014, several studies in Makkah reported increased antimicrobial resistance in *Acinetobacter* (Table 1). For example, in a study conducted between 2004 and 2005, *Acinetobacter* spp. isolates from septicemic patients were found to be highly resistant to a range of antimicrobial agents, with 92% resistant to cefoxitin, 90% resistant to aztreonam, 87% resistant to amoxicillin/clavulanic acid and 77% resistant to ciprofloxacin. However, only 8% of isolates were resistant to piperacillin/tazobactam and 14% were resistant to imipenem [56]. This is consistent with another study conducted in two main tertiary care hospitals in Makkah during 2005–2006, which showed that *A. baumannii* was highly resistant to most antimicrobial agents with rates ranging from 50 to 100%. *A. baumannii* was found to be moderately resistant to imipenem, piperacillin/tazobactam and meropenem, with rates of 15 to 40% [57]. Over 2 years from 2012 to 2014, A total of 107 *A. baumannii* samples were collected from ICU patients at local general hospitals in Makkah and most of the isolates were found to be resistant to cefepime, ceftazidime, and aztreonam [17]. Another hospital-based, matched case–control study confirmed that *A. baumannii* tends to be resistant to several antibiotics [7].

Jeddah is another important city in the country’s western area, with the second largest population after Riyadh [54]. From May 1999 to April 2000, a total of 170 isolates of *A. baumannii* were recovered from clinical specimens of patients as King Abdulaziz University Hospital. *A. baumannii* revealed high rates of resistance to the most tested antimicrobial agents, with the exception of imipenem (15%) [1]. A retrospective study was also conducted over 4 years periods from January 2010 to December 2013 at King Abdulaziz Hospital to analyze the epidemiology and resistance patterns of *A. baumannii*. The prevalence of MDR *A. baumannii* was high throughout this study and increasing from 55% in 2010 to 67% in 2013 [20]. In 2012, a study investigated clinical isolates of hospitalized patients within all the 12 Ministry of Health hospitals in Jeddah *A. baumannii* counted for 17% (754/4404) of the total gram-negative isolates identified, and the resistance rates were 25% for ceftazidime, 27% for ciprofloxacin, 27% for cefepime, 34% for amikacin, 52% for tazocin, 57% for gentamicin and 66% for imipenem, and no resistance to colistin was detected [54]. The high rates of MDR *A. baumannii* observed in this area indicates a need for the careful use of antibiotics, improved infection control strategies and continuous surveillance of antimicrobial susceptibility of this pathogen [1, 20, 54].

The Madinah region includes the second holiest city in Islam (Al-Madinah Al-Monawarah), which lies inland about 400 km north of Makkah. Studies also reported emerging antimicrobial resistance among *A. baumannii* in this region. A 6-month prospective study for the presence of MDR *A. baumannii* infection or colonization on inpatients, health care workers, and environmental sites was done in the ICU of King Fahd Hospital. EL-Ageery et al. [22] found that all of the *A. baumannii* isolates were considered MDR, and were resistant to imipenem, piperacillin–tazobactam, and commonly used antibiotics including cephalosporines, aztreonam and amikacin. These findings are confirmed by another study on 48 CRAB strains isolated from patients with different types of infection either admitted or attending outpatient clinics at a similar hospital [55]. This study demonstrated that all of the isolates were resistant to cefoxitin, imipenem, ceftazidime, and ciprofloxacin. However, there were varying resistant patterns to amoxicillin/clavulanic acid, piperacillin, gentamycin, amikacin, and aztreonam [55]. Implementing proper infection control measures and strict antibiotic policies might be useful in combating the spread of MDR *A. baumannii* in this region [22].

## Eastern area

In the eastern region, many reports demonstrate the existence of CRAB strains in a variety of hospital settings [12, 27, 68] Abdalhamid et al. [12] characterized the occurrence of CRAB isolates in a tertiary care hospital from January 2010 to February 2012, and found that all of the *A. baumannii* strains analysed (n = 141) were susceptible to colistin. The percentage of CRAB isolates was 32.6%, with the highest rates of resistance observed for ceftazidime (85.1%), cefepime (73.8%), and ciprofloxacin (69.5%). The resistance rates were 32.6% for imipenem and 33.3% for both meropenem and piperacillin–tazobactam. These rates are similar to those reported elsewhere in Saudi Arabia and in other neighbouring countries, indicating the spreading of CRAB strains. Other authors evaluated the prevalence of CRAB colonization by isolating intestinal microbiota from ICU patients at two hospitals in Dammam and Khobar from January 2014 to June 2014. Aljindan et al. [27] reported the resistance rates in the CRAB isolates were 100% for ciprofloxacin and cefepime, 62.9% for gentamicin, 51.4% for tigecycline and 40% for amikacin. Moreover, CRAB isolates were significantly (P < 0.05) more resistant to cefepime, amikacin, gentamicin, tigecycline, and ciprofloxacin in comparison to carbapenem susceptible *Acinetobacter* strains. However, CRAB isolates were not significantly more resistant to colistin than carbapenem susceptible isolates (P > 0.05). Another study conducted over a 3-month period from March to May 2014 confirmed these findings. Ten CARB isolates from two tertiary care hospitals were found to be resistant to cefotaxime, ceftazidime, cefepime, and imipenem, meropenem and ciprofloxacin; however, they were all susceptible to colistin [61].

## Southern area

In the southern area of Saudi Arabia, a number of studies have been conducted among clinical isolates of *A. baumannii* from the Aseer and Nagran regions [19, 25, 62, 69, 70]. The amount of antimicrobial resistance among *A. baumannii* continues to rise, and is considered to be a new threat in the Aseer region [69]. *A. baumannii* has been found to be highly resistant to several antimicrobial agents in this region (Table 1). In a study carried out between 2013 and 2014 in Aseer Central Hospital, a referral hospital in the Aseer region, *A. baumannii* has been observed to be highly resistant to cefepime (89.8%) ciprofloxacin (82.9%) and gentamicin (81.5%) [63]. In another study conducted during 2014–2015 in the similar hospital, antimicrobial resistance rates were increased among *A. baumannii* isolates from ICU [19]. Fortunately, colistin remains effective agents against *A. baumannii* isolates in this a study [19]. Consistently, high rates of resistance to the common antimicrobial agents have been reported among *Acinetobacter* isolates from patients at different ICUs in King Abdulla Hospital at Bisha province, northern Aseer region [70].

In the Najran region, a study conducted during a period from October 2012 to March 2013, in a 350-bed tertiary care hospital in Najran city, demonstrated *A. baumannii* isolates that were highly resistant to ceftazidime (91%), trimethoprim/sulfamethoxazole (77.9%), gentamicin (60.3%) and ciprofloxacin 45.6% [25]. These findings indicate the importance of local resistance data for guiding physicians in determining suitable antibiotics for empirical therapy for infections caused by these pathogens [25].

## Northern area

Data regarding the antimicrobial resistance patterns of *A. baumannii* in the northern regions are scarce. A study conducted in the Al Jouf region to determine the prevalence, etiological agents, drug susceptibility pattern and plasmid profile of *A. baumannii* isolates from hospital-acquired infections at Community Hospital. *A. baumannii* demonstrated that 7.1% of isolates were resistant to the most commonly prescribed antimicrobial agents [71]. According to Selim and Hagag [71], this due to the intense use of antimicrobial agents in the hospital, easy availability and indiscriminate use of these drugs outside hospitals, and many antibiotics are available over the counter for self-medication. These problems, coupled with the increased risk of cross-infection among inpatients, are known to account for increasing the circulation of resistant strains.

## Mechanisms of carbapenem resistance

The prevalence of CRAB has recently increased exponentially throughout the world, limiting therapeutic options and increasing the morbidity and mortality rates of infections caused by this pathogen [33, 52, 72]. The resistance of *A. baumannii* to carbapenems is mediated mainly by alterations in the outer membrane proteins, the development of efflux pumps, changes in penicillin-binding proteins, and by the production of different types of beta-lactamases. Beta-lactamases are classified into four classes: A, B, C and, D. Carbapenem resistance is mainly mediated by class B (Metallo-b-lactamases, MBLs), and class D (OXA-type carbapenemases), with class A less frequently implicated [12, 16]. OXA enzymes, encoded by blaOXA genes, can be classified into eight distinct subgroups, of which OXA-23- like, OXA-24-like, OXA-40-like, OXA-51-like, and OXA-58-like and OXA-143-like have been identified in *A. baumannii* [12, 16]. The genes coding these enzymes are regulated by upstream insertion sequences (IS), specifically ISAba1, ISAba2, ISAba3, ISAba9 and IS18. These perform a major role in the expression of blaOXA genes and result in increased resistance to carbapenems [12, 16, 45]. In addition to OXA carbapenemases, transferable MBL families including VIM, IMP, GIM, SIM and NDM enzymes are associated with the resistant phenotype in *A. baumannii* [33, 55].

The co-existence and wide distribution of many clones of CRAB carrying multiple genetic mechanisms of carbapenem-resistance have been documented in Saudi Arabia (Table 2). In a study conducted in hospitals throughout the country, clinical samples were collected from diabetic patients from 2006 to 2007. Nine strains possessed four novel blaOXA-51-like genes encoding b-lactamases, designated OXA-90, OXA-130, OXA-131 and OXA-132, and four strains contained blaOXA-131 with iSAba1 upstream of the gene structure. In the year 2012, Memish et al. [73] characterized the molecular basis of carbapenemase production in carbapenem-resistant gram-negative bacteria isolated from hospitalized patients from Saudi Arabia. OXA-23 were found to be the dominant carbapenemases in *A. baumannii*. Moreover, this was the first study in the country reported the coexistence of blaOXA-23 and blaNDM-1 genes and the presence of OXA-72 and NDM-1 genes in *A. baumannii*. Another study conducted in the gulf council countries including Saudi Arabia reported the most common resistant genes among CRAB isolates were the OXA-23-type, followed by the OXA-40-type (OXA-24-type), and the ISAba1 element upstream of blaOXA-51-type [33]. In the eastern region, Abdalhamid et al. [12] confirmed that blaOXA-23 carbapenemase is the most common gene responsible for resistance. In another study of 83 isolates collected between 2008 and 2012 from specimens of patients at seven major hospitals in the Eastern region, the blaVIM gene was detected in 94% of isolates, while blaOXA-23-like genes were detected in 58% [74]. This fits with another study in Saudi Arabia examining the relationship between the resistance mechanisms *A. baumannii* and whether or not affected patients had diabetes. Alsultan et al. [6] reported the presence of the A blaVIM gene in ~ 95% of isolates; while the blaOXA-23 and blaOXA-40 genes were also prevalent. Diabetic patients were significantly more likely to carry CRAB isolates. In accordance to these findings, a prospective study of CRAB isolates from patients with different types of infection at King Fahd Hospital in Al-Madinah Al-Monawarah demonstrated the presence of the VIM-1 gene in 61.5% of isolates [55]. Moreover, El-Mahdy et al. [61]. found that all of the ten carbapenem-resistant *A. calcoaceticus*–*baumannii* complex isolates from the eastern region harbored blaOXA-23-like and ISAba1, and that 9 of them also carried blaADC. Three out of the ten isolates harbored blaNDM-1 coding for NDM Metallo-b-lactamase (MBL), with the coexistence of blaADC together with either blaGES or blaTEM or both. In the year 2014, a study was conducted in two hospitals in Dammam and Khobar to characterize the molecular mechanisms of 35 CRAB isolates from rectal swab specimens from ICU patients. OXA-23 β-lactamase was detected in all of the carbapenem resistant *A. baumannii*, and no IMP-, VIM-, SPM-, SIM-, GIM, KPC-, and NDM-β-lactamases were detected in these isolates; confirming that OXA-23 is the main 42 mechanism of carbapenem resistance in these isolates [27]. A similar observation was reported in *A. baumannii* isolates from Riyadh. Al-Agamy et al. [52] found that OXA-23 was the most prevalent acquired resistant gene, and Metallo-b-lactamases have no role in carbapenem resistance in the in *A. baumannii* isolates.

**Table 2:** Types of carbapenems resistant genes carried by *A. baumannii* collected from various clinical specimens of patients at Saudi Arabia hospitals

Region	Refs.	Year of sampling	Specimens	Sources of isolates	Setting	No. of carbapenems resistant isolates	Types of carbapenems genes (%)
Across KSA	[37]	2006–2007	Sputum, urine, blood, ulcer	Inpatients and outpatients with DM	Different hospital wards	20	blaOXA-51 (45); blaOXA-131 (20)
Across KSA	[6]	2008–2011	Blood, respiratory, urine, wound	Inpatients	ICUs	196	A blaVIM (93); blaOXA-23 (55); blaOXA-40 (30); ISAba1-blaOXA-51 (4)
Across KSA	[16]	2012	Respiratory, blood, urine, abdomen, skin	Inpatients with DM	ICUs	64	blaVIM (92.2); blaOXA-23 (53.1); blaOXA-40 (29.7); blaSPM (28.1)
Across KSA	[73]	2012	Respiratory, skin, wound, urine	Inpatients	Different hospital wards	79	OXA-51 (100); OXA-23 (97.5); OXA-72 (2.5); NDM-1 (1.2); OXA-23+NDM-1 (1.2)
Aseer	[63]	2013–2014	Tracheal aspirate, sputum, wound and urine	Inpatients	Different hospital wards	56	blaOXA-23 (85.7); blaOXA-40 (5.4); blaOXA-58 (3.6);
Eastern region	[12]	2010–2012	Respiratory, wound, urine	Inpatients and outpatients	Different hospital wards and adult ICU	46	blaOXA-23 (80.4); blaOXA-51 (2.2)
Eastern region	[74]	2008–2012	Various clinical specimens^b^	Inpatients	Different hospital wards	57	blaOXA-23 (58); blaOXA-40 (13); blaVIM (94)
Eastern region	[27]	2014	Rectal swab	Inpatients	ICUs	35	OXA-23 (100)
Eastern region	[61]	2014	Sputum, blood, wound, nasal swab, pus swab	Inpatients and outpatients	Different hospital wards	10	blaOXA-23 (10); blaNDM-1 (30)
Eastern region	[68]	NM	Sputum, throat, abdomen, catheter and endotracheal aspirates	Inpatients	ICUs	5	OXA-23 (100)
Madinah	[55]	NM	Wound, sputum, urine, blood	Inpatients and outpatients	Different hospital wards	48	VIM-1 gene (37.9)
Makkah	[17]	2012–2014	Blood, and skin wound	Inpatients	ICUs	100^a^	OXA-51-like (94); OXA-23-like (91)
Riyadh	[53]	2011	NM	Inpatients	Different hospital wards	55	blaOXA-23 (60.0); blaPER (49.1); blaGES (34.5); blaOXA-24 (3.6)
Riyadh	[50]	2006–2008	Respiratory, wound and burn, blood, urine and other samples	Inpatients	Different hospital wards	253	OXA-23 (100); OXA-51 (100); OXA-58 (1.6)
Riyadh	[18]	2012	Tracheal aspirate	Inpatients	Adults ICU	12	OXA-51 (100); OXA-23 (91.7); OXA-24/40 (8.3)
Riyadh	[2]	2006–2014	Respiratory, wound and burn, blood, urine, and others samples	Inpatients	Different hospital wards	503	OXA-51-like (90.8); PER-1 (76.3)
Riyadh	[52]	2010	Various clinical specimens^b^	Inpatients	Different hospital wards	27	OXA-23 (63); OXA-24/40 (3.7); ADC (96.3); GES-1 (18.5); GES-11 (11); GES-5 (3.7)
Riyadh-Khobar	[33]	2011–2013	Blood, sputum, urine, respiratory, body fluid	Inpatients and outpatients	Different hospital wards	80	OXA-51 (100); OXA-23 (93)

*ICU* intensive care unit, *KSA* Kingdom of Saudi Arabia, *NM* not mentioned

^a^Isolates were positive for extended spectrum beta lactamases

^b^Types of clinical specimens were not specified in the study

During 2011, the distribution of b-lactamase genes among 55 consecutive *A. baumannii* isolates with reduced susceptibility to imipenem collected at Prince Salman Hospital. Al-Agamy et al. [53] indicated that carbapenem resistance was consistently associated with blaOXA-23 or blaOXA-24, whereas low-level resistance was associated with the presence of ESBLs of GES or PER type and/or ISAba1-upregulated blaOXA-51-like. Consistently, Al-Obeid et al. [18] reported that the main mechanism of carbapenem resistance is mediated by class D-OXA-type enzymes (oxa-23 and oxa-24/40) with carbapenemase activity. From 2012 to 2014, ESBL positive *A. baumannii* isolates (n = 100) from the ICUs of local hospitals in Makkah were genotypically characterized. Ninety-four (94%) of isolates carried the carbapenemase gene OXA51-like, and ninety-one (91%) contained OXA23-like. This molecular diversity of ESBL genes contributes to increasing patterns of resistance which indicate the need for regular screening for ESBL-producing *A. baumannii* [17]. Another study identified the genetic diversity of OXA-51-like genes using sequence-based typing (SBT) and multilocus sequence typing (MLST) among MDR *A. baumannii* at a tertiary care hospital. Aly et al. [50] found that SBT demonstrated high OXA-51-like genotypic diversity and showed that all of the isolates were clustered into four main groups: OXA-66 OXA- 69, OXA-132 and other OXA-51-like genes, including OXA-79, -82, -92, -131 and -197. However, more studies are required to explore the molecular mechanisms that confer carbapenem-resistant phenotypes and to investigate the genetic diversity of other OXA-D genes. Another study led by the similar author determined the prevalence and genetic diversity of extended-spectrum beta-lactamase genes, particularly the PER-1 gene among CRAB strains, and the genotyping results of PER-1-like genes showed that 76.3% were positive among MDR *Acinetobacter* isolates. Based on SBT, the majority of these isolates were clustered into three main groups including isolates harboring PER-1: AB11 (bla-PER-1), isolate AB16 (bla-PER-1), and plasmid pAB154 (bla-PER-7) [2].

## Conclusions

Multiple complex factors related to the hospital environment, patient co morbidities, duration of hospital admission, ICU complexity, intercurrent illness and the usage of antimicrobial agents contribute to the spread of *A. baumannii*. Increasing resistance of *A. baumannii* to several classes of antimicrobial agents is a real concern, and has been found to be a great problem in many tertiary referral hospitals in Saudi Arabia. This dilemma has been documented in many parts of the country and may challenge local health authorities. However, data is still scarce in certain local areas as well as in the northern region. The high rates of resistant *A. baumannii* in Saudi Arabia call for comprehensive surveillance programs to understand the origins and extent of the *A. baumannii* problem in depth. However, developing a local antibiogram database coupled with nationwide antimicrobial stewardship and an infection prevention program might help in improving the knowledge of *A. baumannii* resistance patterns. CRAB is widely distributed, and has become a major cause of healthcare-associated infections in the hospitals’ settings. OXA-23 β-lactamase and OXA-51 β-lactamase is a prevalent gene responsible for CRAB, other novel genes such as blaVIM, PER-1-like and GES-5 have been discovered in carbapenem-resistant strains. Research efforts should be focused on the molecular basis of carbapenem resistance; and discovering new therapies for CRAB might contribute to the global effort of combating this pathogen and decreasing the burden of MDR infection.

## References

[1] Khalaf RMF, El Tahawy ATAE (2001). Antibiotic resistance among gram-negative non-fermentative bacteria at a teaching hospital in Saudi Arabia. J Chemother.

[2] Aly MM, Abu Alsoud NM, Elrobh MS, Al Johani SM, Balkhy HH (2016). High prevalence of the PER-1 gene among carbapenem-resistant *Acinetobacter baumannii* in Riyadh, Saudi Arabia. Eur J Clin Microbiol Infect Dis.

[3] Nicasio AM, Kuti JL, Nicolau DP (2008). The current state of multidrug-resistant gram-negative bacilli in North America. Pharmacotherapy.

[4] Maragakis LL, Perl TM (2008). Antimicrobial resistance: *Acinetobacter baumannii*: epidemiology, antimicrobial resistance, and treatment options. Clin Infect Dis.

[5] Poirel L, Bonnin RA, Nordmann P (2011). Genetic basis of antibiotic resistance in pathogenic *Acinetobacter* species. IUBMB Life.

[6] Alsultan AA, Evans BA, Elsayed EA, Al-Thawadi SI, Al-Taher AY, Amyes SGB (2013). High frequency of carbapenem-resistant *Acinetobacter baumannii* in patients with diabetes mellitus in Saudi Arabia. J Med Microbiol.

[7] Al-Gethamy MM, Faidah HS, Adetunji HA, Haseeb A, Ashgar SS, Mohanned TK (2017). Risk factors associated with multi-drug-resistant *Acinetobacter baumannii* nosocomial infections at a tertiary care hospital in Makkah, Saudi Arabia—a matched case–control study. J Int Med Res.

[8] Poirel L, Nordmann P (2006). Carbapenem resistance in *Acinetobacter baumannii*: mechanisms and epidemiology. Clin Microbiol Infect.

[9] Al-Anazi KA, Al-Jasser AM (2014). Infections caused by *Acinetobacter baumannii* in recipients of hematopoietic stem cell transplantation. Front Oncol.

[10] Yezli S, Shibl AM, Memish ZA (2015). The molecular basis of β-lactamase production in gram-negative bacteria from Saudi Arabia. J Med Microbiol.

[11] Ahmed MU, Farooq R, Al-Hawashim N, Ahmed M, Yiannakou N, Sayeed F (2015). Sensitive, resistant and multi-drug resistant *Acinetobacter baumannii* at Saudi Arabia hospital eastern region. Pak J Pharm Sci.

[12] Abdalhamid B, Hassan H, Itbaileh A, Shorman M (2014). Characterization of carbapenem-resistant *Acinetobacter baumannii* clinical isolates in a tertiary care hospital in Saudi Arabia. New Microbiol.

[13] Dhabaan GN, AbuBakar S, Cerqueira GM, Al-Haroni M, Pang SP, Hassan H (2015). Imipenem treatment induces expression of important genes and phenotypes in a resistant *Acinetobacter baumannii* isolate. Antimicrob Agents Chemother.

[14] Senok A, Garaween G, Raji A, Khubnani H, Sing GK, Shibl A (2015). Genetic relatedness of clinical and environmental *Acinetobacter baumannii* isolates from an intensive care unit outbreak. J Infect Dev Ctries.

[15] Zowawi HM, Balkhy HH, Walsh TR, Paterson DL (2013). β-lactamase production in key gram-negative pathogen isolates from the Arabian Peninsula. Clin Microbiol Rev.

[16] Alsultan AA, Aboulmagd E, Evans BA, Amyes SGB (2014). Clonal diversity of *Acinetobacter baumannii* from diabetic patients in Saudi Arabian hospitals. J Med Microbiol.

[17] Alyamani EJ, Khiyami MA, Booq RY, Alnafjan BM, Altammami MA, Bahwerth FS (2015). Molecular characterization of extended-spectrum beta-lactamases (ESBLs) produced by clinical isolates of *Acinetobacter baumannii* in Saudi Arabia. Ann Clin Microbiol Antimicrob.

[18] Al-Obeid S, Jabri L, Al-Agamy M, Al-Omari A, Shibl A (2015). Epidemiology of extensive drug resistant *Acinetobacter baumannii* (XDRAB) at Security Forces Hospital (SFH) in Kingdom of Saudi Arabia (KSA). J Chemother.

[19] Al Bshabshe A, Joseph MRP, Al Hussein A, Haimour W, Hamid ME (2016). Multidrug resistance *Acinetobacter* species at the intensive care unit, Aseer Central Hospital, Saudi Arabia: a one year analysis. Asian Pac J Trop Med.

[20] Al Mobarak MF, Matbuli RM, Meir H, Al Gehani N, Eltoukhy AAM, Al Qureshey KF (2014). Antimicrobial resistance patterns among *Acinetobacter baumannii* isolated from King Abdulaziz Hospital, Jeddah, Saudi Arabia, four-year surveillance study (2010–2013). Egypt J Med Microbiol.

[21] Mah MW, Memish ZA, Cunningham G, Bannatyne RM (2001). Outbreak of *Acinetobacter baumannii* in an intensive care unit associated with tracheostomy. Am J Infect Control.

[22] El-Ageery SM, Abo-Shadi MA, Alghaithy AA, Ahmad MA, Alsharif NH, Alharbi SA (2012). Epidemiological investigation of nosocomial infection with multidrug-resistant *Acinetobacter baumannii*. Eur Rev Med Pharmacol Sci.

[23] El-Saed A, Balkhy HH, Al-Dorzi HM, Khan R, Rishu AH, Arabi YM (2013). Acinetobacter is the most common pathogen associated with late-onset and recurrent ventilator-associated pneumonia in an adult intensive care unit in Saudi Arabia. Int J Infect Dis.

[24] Praczko K, Kostka T (2006). Infections in the elderly. Part I. Etiology and pathogenesis. Wiad Lek.

[25] Asaad AM, Al-Ayed MSZ, Qureshi MA (2013). Emergence of unusual nonfermenting gram-negative nosocomial pathogens in a Saudi hospital. Jpn J Infect Dis.

[26] Ballouz T, Aridi J, Afif C, Irani J, Lakis C, Nasreddine R (2017). Risk factors, clinical presentation, and outcome of *Acinetobacter baumannii* bacteremia. Front Cell Infect Microbiol.

[27] Aljindan R, Bukharie H, Alomar A, Abdalhamid B (2015). Prevalence of digestive tract colonization of carbapenem-resistant *Acinetobacter baumannii* in hospitals in Saudi Arabia. J Med Microbiol.

[28] Bukhari SZ, Hussain WM, Banjar AA, Fatani MI, Karima TM, Ashshi AM (2012). Application of ventilator care bundle and its impact on ventilator associated pneumonia incidence rate in the adult intensive care unit. Saudi Med J.

[29] Leangapichart T, Gautret P, Griffiths K, Belhouchat K, Memish Z, Raoult D (2016). Acquisition of a high diversity of bacteria during the hajj pilgrimage, including *Acinetobacter baumannii* with blaOXA-72 and *Escherichia coli* with blaNDM-5 carbapenemase genes. Antimicrob Agents Chemother.

[30] Joly-Guillou ML (2005). Clinical impact and pathogenicity of *Acinetobacter*. Clin Microbiol Infect.

[31] Khan MA, Mahomed MF, Ashshi AM, Faiz A (2012). Drug resistance patterns of *Acinetobacter baumannii* in Makkah, Saudi Arabia. Pak J Med Res.

[32] Nurain AM, Bilal NE, Ibrahim ME (2015). Asian Pacific Journal of Tropical Biomedicine recovered from cancer patients and hospital environments. Asian Pac J Trop Biomed.

[33] Zowawi HM, Sartor AL, Sidjabat HE, Balkhy HH, Walsh TR, Al Johani SM (2015). Molecular epidemiology of carbapenem-resistant *Acinetobacter baumannii* isolates in the Gulf Cooperation Council States: dominance of OXA-23-type producers. J Clin Microbiol.

[34] Al-Anazi KA, Abdalhamid B, Alshibani Z, Awad K, Alzayed A, Hassan H (2012). *Acinetobacter baumannii* septicemia in a recipient of an allogeneic hematopoietic stem cell transplantation. Case Rep Transplant.

[35] Al-Otaibi FE, Bukhari EE, Badr M, Alrabiaa AA (2016). Prevalence and risk factors of gram-negative bacilli causing blood stream infection in patients with malignancy. Saudi Med J.

[36] Luo G, Spellberg B, Gebremariam T, Bolaris M, Lee H, Fu Y (2012). Diabetic murine models for *Acinetobacter baumannii* infection. J Antimicrob Chemother.

[37] Alsultan AA, Hamouda A, Evans BA, Amyes SGB (2009). *Acinetobacter baumannii*: emergence of four strains with novel bla OXA-51-like genes in patients with diabetes mellitus. J Chemother.

[38] Rana MA, El Rahman BA, Mady AF, Al Odat M, AlHarthy A, Ramadan OES (2014). Intra-pleural colistin methanesulfonate therapy for pleural infection caused by carbapenem-resistant *Acinetobacter Baumannii*: a successful case report. Infect Dis Rep.

[39] Mairghani M, Elmusharaf K, Patton D, Burns J, Eltahir O, Jassim G (2017). The prevalence and incidence of diabetic foot ulcers among five countries in the Arab world: a systematic review. J Wound Care.

[40] Shakil SKA (2010). Infected foot ulcers in male and female diabetic patients: a clinico-bioinformative study. Ann Clin Microbiol Antimicrob.

[41] Khan DM, Moosabba MS, Rao IV (2018). Changing antibiogram profile of *Acinetobacter baumannii* in diabetic and non-diabetic foot ulcer infections. J Clin Diagn Res.

[42] Lemos EV, de la Hoz FP, Einarson TR, Mcghan WF, Quevedo E, Castañeda C (2014). Carbapenem resistance and mortality in patients with *Acinetobacter baumannii* infection: systematic review and meta-analysis. Clin Microbiol Infect.

[43] Kandemir Ö, Akbay E, Şahin E, Milcan A, Gen R (2007). Risk factors for infection of the diabetic foot with multi-antibiotic resistant microorganisms. J Infect.

[44] Bertrand X, Dowzicky MJ (2012). Antimicrobial susceptibility among gram-negative isolates collected from intensive care units in North America, Europe, the Asia-Pacific Rim, Latin America, the Middle East, and Africa between 2004 and 2009 as part of the tigecycline evaluation and survei. Clin Ther.

[45] Howard A, O’Donoghue M, Feeney A, Sleator RD (2012). *Acinetobacter baumannii*. Clin Microbiol Rev.

[46] Baadani AM, Thawadi SI, El-khizzi NA, Omrani AS (2013). *Acinetobacter baumannii* clinical isolates from 2 hospitals. Saudi Med J.

[47] Said KB, Al-Jarbou AN, Alrouji M, Al-Harbi HO (2014). Surveillance of antimicrobial resistance among clinical isolates recovered from a tertiary care hospital in Al Qassim, Saudi Arabia. Int J Health Sci (Qassim).

[48] Assiri AM, Choudhry AJ, Alsaleh SS, Alanazi KH, Alsaleh SS (2014). Evaluation of infection prevention and control programmes (IPC), and assessment tools for IPC-programmes at MOH-health facilities in Saudi Arabia. Open J Nurs.

[49] Abdallah E, Ahamed F (2015). Antibiotic susceptibility patterns of some clinical isolates from Al-Rass General Hospital. Int J Biosci.

[50] Aly M, Tayeb HT, Al Johani SM, Alyamani EJ, Aldughaishem F, Alabdulkarim I (2014). Genetic diversity of OXA-51-like genes among multidrug-resistant *Acinetobacter baumannii* in Riyadh, Saudi Arabia. Eur J Clin Microbiol Infect Dis.

[51] Saeed NK, Kambal AM, El-Khizzi NA (2010). Antimicrobial-resistant bacteria in a general intensive care unit in Saudi Arabia. Saudi Med J.

[52] Al-Agamy MH, Jeannot K, El-Mahdy TS, Shibl AM, Kattan W, Plesiat P (2017). First detection of GES-5 carbapenemase-producing *Acinetobacter baumannii* isolate. Microb Drug Resist.

[53] Al-Agamy MH, Shibl AM, Ali MS, Khubnani H, Radwan HH, Livermore DM (2014). Distribution of beta-lactamases in carbapenem-non-susceptible *Acinetobacter baumannii* in Riyadh, Saudi Arabia. J Glob Antimicrob Resist.

[54] Halwani MA (2017). Basic and applied research. Albaha Univ J Basic Appl Sci.

[55] El-Ageery SM, Al-Hazmi SS (2014). Microbiological and molecular detection of VIM-1 metallo beta lactamase-producing *Acinetobacter baumannii*. Eur Rev Med Pharmacol Sci.

[56] Asghar AH (2006). Frequency and antimicrobial susceptibility patterns of bacterial pathogens isolated from septicemic patients in Makkah hospitals. Saudi Med J.

[57] Asghar AH, Faidah HS (2009). Frequency and antimicrobial susceptibility of gram-negative bacteria isolated from 2 hospitals in Makkah, Saudi Arabia. Saudi Med J.

[58] Haseeb A, Faidah HS, Bakhsh AR, Al Malki WH, Elrggal ME, Saleem F (2016). Antimicrobial resistance among pilgrims: a retrospective study from two hospitals in Makkah, Saudi Arabia. Int J Infect Dis.

[59] Al-Tawfiq JA, Mohandhas TX (2007). Prevalence of antimicrobial resistance in *Acinetobacter calcoaceticus*–*baumannii* complex in a Saudi Arabian Hospital. Infect Control Hosp Epidemiol.

[60] Abdalhamid B, Elhadi N, Alabdulqader N, Alsamman K, Aljindan R (2016). Rates of gastrointestinal tract colonization of carbapenem-resistant Enterobacteriaceae and *Pseudomonas aeruginosa* in hospitals in Saudi Arabia. New Microbes New Infect.

[61] El-Mahdy TS, Al-Agamy MH, Al-Qahtani AA, Shibl AM (2017). Detection of bla OXA-23-like and bla NDM-1 in *Acinetobacter baumannii* from the Eastern Region, Saudi Arabia. Microb Drug Resist.

[62] Abdalla NM, Osman AA, Haimour WO, Sarhan MAA, Mohammed MN, Zyad EM (2013). Antimicrobial susceptibility pattern in nosocomial infections caused by *Acinetobacter* species in Asir Region, Saudi Arabia. Pak J Biol Sci PJBS.

[63] Elabd FM, Al-Ayed MSZ, Asaad AM, Alsareii SA, Qureshi MA, Musa HA-A (2015). Molecular characterization of oxacillinases among carbapenem-resistant *Acinetobacter baumannii* nosocomial isolates in a Saudi hospital. J Infect Public Health.

[64] Kengkla K, Kongpakwattana K, Saokaew S, Apisarnthanarak A, Chaiyakunapruk N (2018). Comparative efficacy and safety of treatment options for MDR and XDR *Acinetobacter baumannii* infections: a systematic review and network meta-analysis. J Antimicrob Chemother.

[65] Somily AM, Absar MM, Arshad MZ, Al Aska AI, Shakoor ZA, Fatani AJ (2012). Antimicrobial susceptibility patterns of multidrug-resistant *Pseudomonas aeruginosa* and *Acinetobacter baumannii* against carbapenems, colistin, and tigecycline. Saudi Med J.

[66] Lakshmana Gowda K, Marie MAM, Al-Sheikh YA, John J, Gopalkrishnan S, Chikkabidare Shashidhar P (2014). A 6-year surveillance of antimicrobial resistance patterns of *Acinetobacter baumannii* bacteremia isolates from a tertiary care hospital in Saudi Arabia during 2005–2010. Libyan J Med.

[67] Al Johani SM, Akhter J, Balkhy H, El-Saed A, Younan M, Memish Z (2010). Prevalence of antimicrobial resistance among gram-negative isolates in an adult intensive care unit at a tertiary care center in Saudi Arabia. Ann Saudi Med.

[68] Sami M, Rashed A (2018). Molecular characterization and antibiotic susceptibility pattern of *Acinetobacter baumannii* isolated in intensive care unit patients in Al-Hassa, Kingdom of Saudi Arabia. Int J Appl Basic Med Res.

[69] Almaghrabi MK, Joseph MRP, Assiry MM, Hamid ME (2018). Multidrug-resistant Acinetobacter baumannii : an emerging health threat in Aseer Region, Kingdom of Saudi Arabia. Can J Infect Dis Med Microbiol.

[70] Ibrahim ME (2018). High antimicrobial resistant rates among gram-negative pathogens in intensive care units. A retrospective study at a tertiary care hospital in Southwest Saudi Arabia. Saudi Med J.

[71] Selim SA, Hagag NI (2013). Analysis of plasmids and restriction fragment length polymorphisms of *Acinetobacter baumannii* isolated from Hospitals-AL Jouf Region-KSA. Proc World Acad Sci Eng Technol.

[72] Lopes BS, Al-Agamy MH, Ismail MA, Shibl AM, Al-Qahtani AA, Al-Ahdal MN (2015). The transferability of blaOXA-23 gene in multidrug-resistant *Acinetobacter baumannii* isolates from Saudi Arabia and Egypt. Int J Med Microbiol.

[73] Memish ZA, Assiri A, Almasri M, Roshdy H, Hathout H, Kaase M (2015). Molecular characterization of carbapenemase production among gram-negative bacteria in Saudi Arabia. Microb Drug Resist.

[74] Al-Sultan AA, Evans BA, Aboulmagd E, Al-Qahtani AA, Bohol MFF, Al-Ahdal MN (2015). Dissemination of multiple carbapenem-resistant clones of *Acinetobacter baumannii* in the Eastern District of Saudi Arabia. Front Microbiol.

